# Basal Ganglia Iron Content Increases with Glioma Severity Using Quantitative Susceptibility Mapping: A Potential Biomarker of Tumor Severity

**DOI:** 10.3390/tomography8020065

**Published:** 2022-03-15

**Authors:** Thomas P. Reith, Melissa A. Prah, Eun-Jung Choi, Jongho Lee, Robert Wujek, Mona Al-Gizawiy, Christopher R. Chitambar, Jennifer M. Connelly, Kathleen M. Schmainda

**Affiliations:** 1 Medical College of Wisconsin, Biophysics, 8701 Watertown Plank Rd., Milwaukee, WI 53226, USA; treith@mcw.edu (T.P.R.); mprah@mcw.edu (M.A.P.); malgizawiy@mcw.edu (M.A.-G.); cchitamb@mcw.edu (C.R.C.); 2 Department of Electrical and Computer Engineering, Seoul National University, Seoul 08826, Korea; karaedduk@hanmail.net (E.-J.C.); jonghoyi@snu.ac.kr (J.L.); 3 Medical College of Wisconsin, Biomedical Engineering, Marquette University, 1515 W. Wisconsin Ave., Milwaukee, WI 53233, USA; rwujek@mcw.edu; 4 Medical College of Wisconsin, Hematology & Oncology, 8701 Watertown Plank Rd., Milwaukee, WI 53226, USA; 5 Medical College of Wisconsin, Neurology & Neurosurgery, 8701 Watertown Plank Rd., Milwaukee, WI 53226, USA; jconnelly@mcw.edu; 6 Medical College of Wisconsin, Radiology, 8701 Watertown Plank Rd., Milwaukee, WI 53226, USA

**Keywords:** quantitative susceptibility mapping (QSM), basal ganglia (BG), MRI, brain tumor iron, glioma

## Abstract

Background and Purpose: Gliomas have been found to alter iron metabolism and transport in ways that result in an expansion of their intracellular iron compartments to support aggressive tumor growth. This study used deep neural network trained quantitative susceptibility mapping to assess basal ganglia iron concentrations in glioma patients. Materials and Methods: Ninety-two patients with brain lesions were initially enrolled in this study and fifty-nine met the inclusion criteria. Susceptibility-weighted images were collected at 3.0 T and used to construct quantitative susceptibility maps via a deep neural network-based method. The regions of interest were manually drawn within basal ganglia structures and the mean voxel intensities were extracted and averaged across multiple slices. One-way ANCOVA tests were conducted to compare the susceptibility values of groups of patients based on tumor grade while controlling for age, sex, and tumor type. Results: The mean basal ganglia susceptibility for patients with grade IV tumors was higher than that for patients with grade II tumors (*p* = 0.00153) and was also higher for patients with grade III tumors compared to patients with grade II tumors (*p* = 0.020), after controlling for age, sex, and tumor type. Patient age influenced susceptibility values (*p* = 0.00356), while sex (*p* = 0.69) and tumor type (*p* = 0.11) did not. Conclusions: The basal ganglia iron content increased with glioma severity. Basal ganglia iron levels may thus be a useful biomarker in glioma prognosis and treatment, especially with regard to iron-based cancer therapies.

## 1. Introduction

Iron is a double-edged sword in human metabolism: it is necessary for life, yet toxic in excess amounts [1]. As an avid participant in redox reactions, iron is an essential cofactor for enzymes comprising the mitochondrial electron transport chain and is thus needed for normal cellular replication and growth [2]. Yet this same redox reactivity allows iron to accelerate the formation of free radical species, which cause irreparable—and potentially mutagenic—damage to DNA and cellular membranes [3].

Recent research interest has focused on the role of iron in cancer pathogenesis. Epidemiological studies in the 1980s first associated high body iron stores with increased cancer risk [4,5,6] and subsequent work has revealed two major mechanisms that explain this relationship. First, the oxidative stress and DNA damage that are induced by excess iron contribute to spontaneous oncogenesis [7]. Second, cancer cells demonstrate an increased dependency on iron, which is needed to fuel their rapid growth and proliferation [8]. Accordingly, such cells alter iron import, export, and storage pathways in ways that result in an expansion of an intracellular iron “pool” to support iron-dependent processes that are increased or activated in malignancy [9]. This reprogramming of iron metabolism in the tumor and its microenvironment is thought to be a critical component of tumor cell survival and growth [9,10].

Recent work has revealed these mechanisms at work in the pathophysiology of gliomas: primary brain tumors that arise from the supportive glial cells that surround and protect neurons. Glioblastomas express high amounts of transferrin receptor 1, which is the main mediator of cellular iron uptake [11], and may also express a second transferrin receptor (transferrin receptor 2) that is not present in normal tissue [12]. Moreover, glioblastomas display increased amounts of ferritin, which is the iron storage protein, and may epigenetically upregulate the production of transferrin itself [13]. The pathways of iron uptake and metabolism are thus promising targets for glioblastoma treatment [14]; indeed, disrupting iron homeostasis has been shown to slow tumor proliferation [15].

Historically, brain iron concentrations have been assessed via postmortem histological examination [16,17]. Recent advances in MRI technology, however, now make in vivo detection feasible [18]. The magnetic susceptibility of a material is a physical property that specifies its degree of internal magnetism in response to an applied magnetic field. The majority of biological materials—such as water, fat, and calcium—are weakly diamagnetic and, therefore, have very small negative susceptibility values. Due to its unpaired electron, however, ferric iron is highly paramagnetic; since tissue iron is predominately stored as ferritin complexes, any increases in the bulk magnetic susceptibility of gray matter are thought to reflect iron deposition [19,20]. The most promising technique for detecting variations in tissue iron is quantitative susceptibility mapping (QSM), which reconstructs the magnetic susceptibility of tissue from gradient echo phase sequence data [21]. The validity of QSM in assessing brain iron concentrations has been confirmed by a number of studies [22,23,24].

We hypothesized that in glioma patients, the increased iron trafficking could extend beyond neoplastic tissue into healthy brain regions. In this study, we used a recently developed deep neural network trained QSM method (QSMnet+ [25,26]) to investigate basal ganglia (BG) iron concentrations in patients with gliomas.

## 2. Materials and Methods

***Subjects***: All participants provided written informed consent according to the institutional review board policy in this Health Insurance Portability and Accountability Act compliant study. Consecutive subjects with brain lesions and susceptibility-weighted imaging collected at our Institution between February 2016 and June 2019 were considered for inclusion in this retrospective study. Exclusion criteria were limited to poor image quality and non-glial or unknown tumor types. A neuropathologist provided a diagnosis for all tumors based on 2016 World Health Organization classification criteria [27].

***Image Collection and Processing***: All MRI exams were performed on a 3.0T MRI system (GE Healthcare, Milwaukee, WI, USA). The data obtained included pre- and post-contrast T1W, T2W, FLAIR, and SWI with acquisition times of 1.7, 2.0, 2.1, 2.1, and 2.1 min, respectively. For the T1W images, a spin echo acquisition was used with: TE = 1.8–1.9 ms, TR = 5.8 ms, flip angle = 10°, and an acquisition matrix of 256 × 192 with a FOV of 220 × 220 mm. A 0.1 mmol/kg intravenous bolus injection of gadobutrol (Gadavist; Bayer Schering Pharma, Berlin, Germany) was administered before the post-contrast images were collected using the same pre-contrast T1W spin echo imaging parameters. For the T2W images, a gradient echo acquisition was used with: TE = 87.9–88.6 ms, TR = 3987–4746 ms, flip angle = 111°, and an acquisition matrix of 416 × 416 with a FOV of 220 × 220 mm^2^. The FLAIR imaging was collected with: TE = 126.3–127.5 ms, TR = 9000 ms, TI = 2250 ms, flip angle = 111°, and an acquisition matrix of 352 × 224 with a FOV of 220 × 220 mm^2^. To collect the SWI imaging, a multi-echo gradient echo acquisition was used with: TE = 13.0, 16.7, 20.4, 24.1, 27.8, 31.5, and 35.2 ms, TR = 38.9–39.1 ms, flip angle = 15°, and an acquisition matrix of 288 × 224 with a FOV of 220 × 220 mm^2^.

To enable the construction of the QSM maps, SWI information was directly saved as k-space data. Each echo’s k-space data were read and then transformed into the image domain. Using coil sensitivities, ASSET unaliasing was performed. The complex unaliased images were separated into real and imaginary parts with gradient warping correction applied separately. Phase images were unwrapped using Laplacian-based unwrapping and the background field was removed using the V-SHARP algorithm [28]. The QSM maps were created using a deep neural network trained method (QSMnet+ [25,26]) and then re-oriented to their starting orientation using the original phase images. The QSM maps were then affinely co-registered to the post-contrast T1W images using FMRIB’s Linear Image Registration Tool (FLIRT; http://fsl.fmrib.ox.ac.uk/fsl/fslwiki/FLIRT (accessed on 9 January 2022)) [29,30,31]. Quantitative delta T1 maps [32] were created from the differences in the standardized pre- and post-contrast T1W images using IB Delta Suite^TM^ software (Imaging Biometrics, Elm Grove, WI, USA).

**Figure 1:**
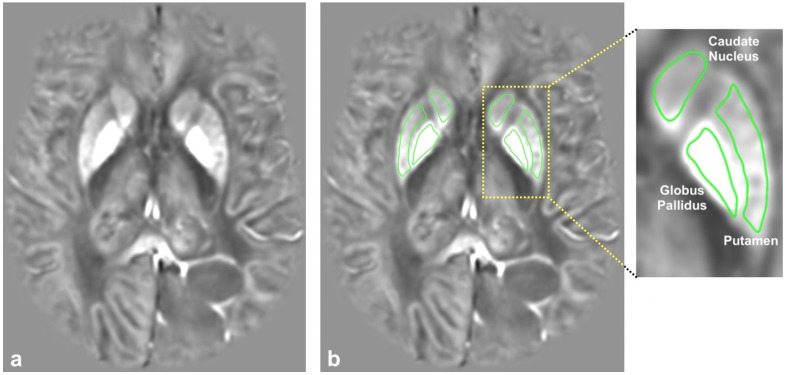
(**a**) A representative QSM image generated with QSMnet+ and (**b**) the same QSM image with the regions of interest (ROI) outlines. An enlargement of the left basal ganglia shows the labeled ROIs for the caudate nucleus, putamen, and globus pallidus.

***Image and Statistical Analysis***: All image analyses were performed using the Horos medical imaging viewer (http://horosproject.org/ (accessed on 9 January 2022)). ROIs for the caudate, putamen, and globus pallidus were manually drawn onto the individual slices of the QSM images (Figure 1). The mean voxel intensities for all ROIs were averaged across multiple slices to obtain one QSM value per tissue region per patient. The resulting QSM values for the caudate, putamen, and globus pallidus were further averaged to obtain one overall BG (basal ganglia) QSM value for each patient.

One-way analysis of covariance (ANCOVA) tests were conducted to compare the BG QSM values of the groups of patients based on tumor grade while controlling for age, sex, and tumor type. The normality of the residuals was assessed using the Shapiro–Wilk test and the homogeneity of variances was established using Levene’s test.

## 3. Results

A total of 92 subjects were enrolled in this study. Of these, 12 were excluded due to having non-glial or unknown tumor types, 20 were excluded because the QSM reconstruction process failed, and 1 was excluded because of significant BG distortion resulting from tumor mass effect. After these exclusions, the cohort of subjects that was analyzed consisted of 59 patients (31 males and 28 females) who had been diagnosed with glioma (44 astrocytoma: 7 grade II, 12 grade III, and 25 grade IV; 14 oligodendroglioma: 8 grade II and 6 grade III; 1 gliosarcoma), ranging from 20 to 84 years old (median = 51) for males and 22 to 83 years old (median = 49.5) for females.

**Figure 2:**
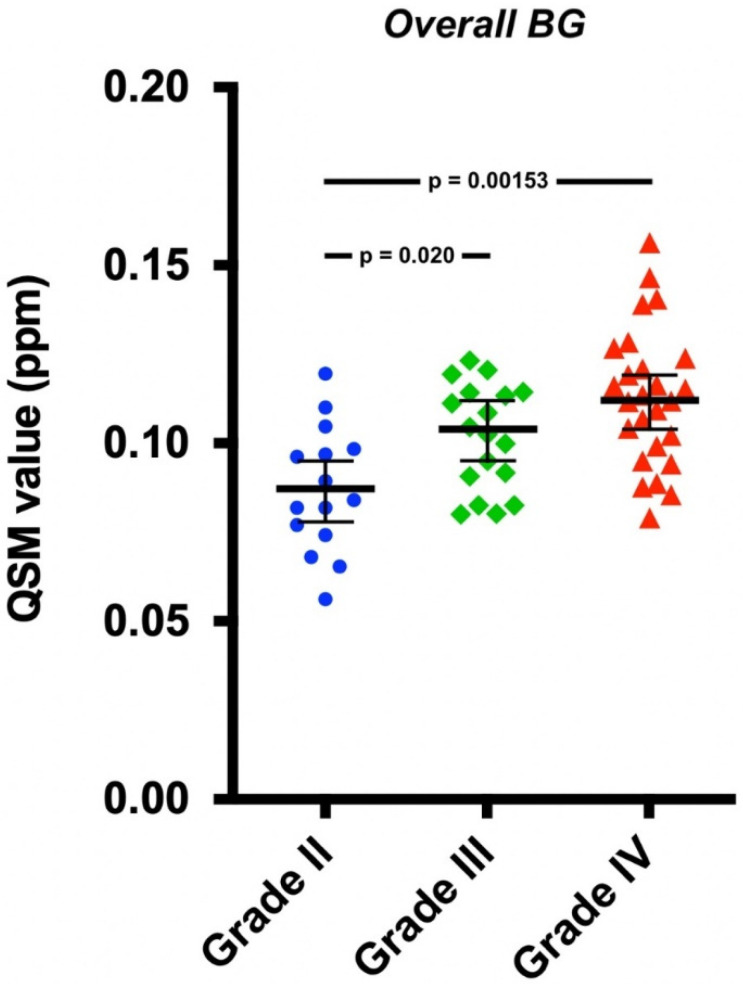
The overall basal ganglia QSM values plotted with covariate adjusted means and standard errors of the means. The means were adjusted for age, sex, and tumor type. The differences were statistically significant between grades II and III and between grades II and IV.

Significant differences between the overall mean BG QSM values of the tumor grades were observed after adjusting for age, sex, and tumor type (F(2,53) = 7.35, *p* = 0.00152) (Figure 2). Post hoc tests showed that QSM values were significantly higher both in patients with grade IV tumors compared to those with grade II tumors (*p* = 0.00153) and in patients with grade III tumors compared to those with grade II tumors (*p* = 0.020). There were no significant differences between the QSM values of grade III and IV tumors (*p* = 0.57). Age was a significant covariate (F(1,53) = 9.31, *p* = 0.00356), while tumor type (F(1,53) = 2.69, *p* = 0.11) and sex (F(1,53) = 0.16, *p* = 0.69) were not.

**Figure 3:**
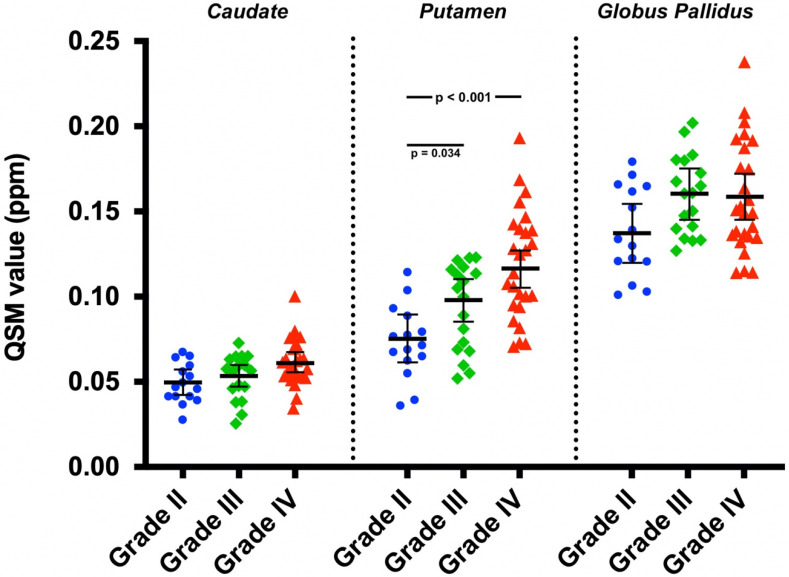
The region-specific basal ganglia QSM values plotted with covariate adjusted means and standard errors of the means. The differences were statistically significant between grades II and III and between grades II and IV for the putamen. The differences for the caudate and globus pallidus were not statistically significant.

The ANCOVA analysis also revealed significant differences between the QSM values of different tumor grades for the putamen (F(2,53) = 8.75, *p* < 0.001) (Figure 3). Post hoc tests showed that QSM values were significantly higher both in patients with grade IV tumors compared to those with grade II tumors (*p* < 0.001) and in patients with grade III tumors compared to those with grade II tumors (*p* = 0.034). There were no significant differences between the putamen QSM values for grade III and IV tumors (*p* = 0.14). No significant differences between the adjusted mean QSM values of different tumor grades were observed for the caudate (F(2,53) = 2.33, *p* = 0.11) or the globus pallidus (F(2,53) = 2.72, *p* = 0.075).

After adjusting for age, sex, and tumor grade, no significant differences were found between the overall mean BG QSM values of astrocytoma or oligodendroglioma (F(1,54) = 3.06, *p* = 0.086).

Table 1 and Table 2 report the covariate adjusted mean QSM values for individual BG regions. Table 3 reports the unadjusted values.

## 4. Discussion

This study demonstrated the tumor severity-related differences in BG iron content in glioma patients. The results were consistent with a previous preliminary report that demonstrated higher BG iron levels in patients with glioblastoma than in patients with lower grade glioma [33]. The results also agreed with previously reported T2 shortening in the BG and thalamus of 23 patients in with both untreated and recurrent brain tumors compared to healthy controls [34]. The T2 shortening was suggestive of an increased iron concentration that was unaffected by treatment.

Regardless of tumor grade, the globus pallidus consistently had the highest susceptibility, followed by the putamen and caudate nucleus. These differences were consistent with previous studies analyzing BG iron content in both healthy patients and those with a neurodegenerative disease [16,24,35]. For example, the use of QSM in healthy brains was validated by using a comparison to the published estimates of regional brain iron concentrations from postmortem and in vivo data [24]. QSM yielded the same rank ordering of iron concentration by brain structure, with the lowest in white matter and the highest in the globus pallidus, as well as yielding the expected age-related changes. QSM also proved more sensitive than R2* in assessing changes in brain iron concentration levels in gray matter nuclei for patients with schizophrenia [36], multiple sclerosis [37,38], and Parkinson’s disease [39,40].

A natural question raised by our findings is whether the increased BG iron is a direct consequence of tumor development or the BG iron content is suggestive of an iron-rich environment that may promote greater tumor growth or aggression. Paraneoplastic syndromes are a known consequence of many tumors, and glioblastomas in particular may upregulate the production of transferrin [13]. When increased tumor iron trafficking occurs, it is reasonable to assume that this iron may also deposit in nearby structures. In fact, it was hypothesized [34] that an increased iron content in the basal ganglia may represent a protective process serving to eliminate excessive ferrous ions from the tissue to provide protection from oxidative stress. On the other hand, high levels of both serum [41] and dietary [42] iron are linked to an increased risk of cancer. Therefore, it is possible that some other systemic process leading to increased body iron may contribute to both carcinogenesis and BG deposition. Future research should investigate whether the increased BG iron levels appear before, during or after tumor initiation and growth.

Another question raised regards the mechanism of iron delivery to the BG. Since we observed no contrast agent enhancement in the BG on the quantitative delta T1 maps, the blood–brain barrier was presumably intact and the direct transfer of iron between plasma and tissue was unlikely. However, previously reported elevated levels of ferritin in the cerebrospinal fluid of glioblastoma patients [43] suggest CSF as an alternative means of iron transport to neural tissue.

Potential confounding factors in this study included age and sex. The associations between age and brain iron are well documented, with progressive iron accumulation in the BG accompanying normal aging [35]. Moreover, the incidence of glioblastoma increases exponentially with age [44]. Together, these factors may explain the significant covariance of age in our analysis. Sex, however, was not a significant covariate. Since recent evidence has demonstrated lower levels of BG iron in both pre- and post-menopausal women compared to men [45,46], it is noteworthy that the BG iron accumulation in this study appeared to be independent of the subject’s biological sex.

Although the differences between tumor grades were significant for the entire BG, the putamen was the only individual region for which they were significant. The adjusted QSM values for the globus pallidus were also slightly higher for patients with grade III tumors than for patients with grade IV tumors. These observations may be due to the small sample size or additional confounding factors affecting BG iron content that we did not take into account. It is also possible that the putamen alone may better reflect tumor severity than other BG regions. This hypothesis is supported by another recent study in which QSM was used to measure brain iron deposition in patients with type II diabetes mellitus. While decreases in susceptibility were noted for the deep gray matter nuclei of patients compared to healthy controls, the change was only significant for the putamen [47]. While an exact mechanism for this difference is not known, it was concluded that iron levels in the putamen best reflected iron overload injury to the central nervous system.

Overall, there seemed to be a clear trend of increased QSM values associated with more aggressive tumors, suggesting that QSM can be used as an independent measure of tumor aggression and/or may help to further elucidate the role of iron metabolism in brain cancer. Future studies involving larger numbers of patients should address these points. 

A final limitation of our study was that no healthy individuals were included due to the lack of available QSM data. Directly comparing the BG QSM values of patients with and without tumors is a logical next step and should be addressed in future work.

## 5. Conclusions

In this study, we showed that basal ganglia iron content increased with glioma severity. These results demonstrate that in patients with gliomas, increased iron trafficking is not limited to neoplastic tissue but may also occur in healthy brain regions. Basal ganglia iron levels may thus be a useful biomarker in glioma prognosis and treatment, especially with regards to iron-based cancer therapies.

## Figures and Tables

**Table 1:** The covariate adjusted mean QSM values for patients with tumors of grades II, III, and IV.

	Covariate Adjusted Mean QSM Value (95% CI) (ppm)		
	Grade II	Grade III	Grade IV
Caudate	0.050 (0.042, 0.057)	0.053 (0.047, 0.060)	0.061 (0.055, 0.067)
Putamen	0.075 (0.061, 0.089)	0.098 (0.085, 0.110)	0.116 (0.105, 0.127)
Globus pallidus	0.137 (0.120, 0.154)	0.160 (0.145, 0.175)	0.159 (0.145, 0.172)
Overall BG	0.087 (0.078, 0.096)	0.104 (0.095, 0.112)	0.112 (0.104, 0.119)

**Table 2:** The covariate adjusted mean QSM values for patients with astrocytoma and oligodendroglioma.

	Covariate Adjusted Mean QSM Value (95% CI) (ppm)
**Astrocytoma**	
Caudate	0.055 (0.051, 0.059)
Putamen	0.096 (0.089, 0.104)
Globus pallidus	0.151 (0.142, 0.160)
Overall BG	0.101 (0.096, 0.106)
**Oligodendroglioma**	
Caudate	0.059 (0.051, 0.066)
Putamen	0.112 (0.097, 0.126)
Globus pallidus	0.162 (0.144, 0.180)
Overall BG	0.111 (0.101, 0.121)

**Table 3:** The unadjusted basal ganglia QSM values for patients with tumors of grades II, III, and IV.

	Mean QSM Value ± SD (ppm)		
	Grade II	Grade III	Grade IV
**All Patients/Tumor Types**			
Caudate	0.049 ± 0.012	0.052 ± 0.013	0.062 ± 0.014
Putamen	0.073 ± 0.021	0.094 ± 0.025	0.119 ± 0.032
Globus pallidus	0.138 ± 0.026	0.160 ± 0.023	0.158 ± 0.032
Overall BG	0.087 ± 0.018	0.102 ± 0.015	0.113 ± 0.019
**Male**			
Caudate	0.051 ± 0.011	0.051 ± 0.013	0.0611 ± 0.0080
Putamen	0.073 ± 0.024	0.094 ± 0.026	0.116 ± 0.025
Globus pallidus	0.137 ± 0.029	0.157 ± 0.021	0.164 ± 0.038
Overall BG	0.087 ± 0.018	0.101 ± 0.016	0.114 ± 0.017
**Female**			
Caudate	0.048 ± 0.013	0.057 ± 0.012	0.062 ± 0.016
Putamen	0.074 ± 0.020	0.094 ± 0.029	0.121 ± 0.036
Globus pallidus	0.140 ± 0.025	0.169 ± 0.029	0.155 ± 0.030
Overall BG	0.087 ± 0.018	0.1066 ± 0.0088	0.113 ± 0.021
**Astrocytoma**			
Caudate	0.046 ± 0.013	0.050 ± 0.015	0.062 ± 0.014
Putamen	0.067 ± 0.024	0.084 ± 0.025	0.119 ± 0.032
Globus pallidus	0.131 ± 0.026	0.157 ± 0.023	0.158 ± 0.032
Overall BG	0.081 ± 0.020	0.097 ± 0.015	0.113 ± 0.019
**Oligodendroglioma**			
Caudate	0.052 ± 0.010	0.0562 ± 0.0074	–
Putamen	0.079 ± 0.018	0.1139 ± 0.0086	–
Globus pallidus	0.144 ± 0.027	0.166 ± 0.023	–
Overall BG	0.092 ± 0.015	0.1120 ± 0.0094	–

## Data Availability

Anonymized data can be made available upon request.
